# Antimicrobial Resistance and Inorganic Nanoparticles

**DOI:** 10.3390/ijms222312890

**Published:** 2021-11-29

**Authors:** Andrea-Sarahí Balderrama-González, Hilda-Amelia Piñón-Castillo, Claudia-Adriana Ramírez-Valdespino, Linda-Lucila Landeros-Martínez, Erasmo Orrantia-Borunda, Hilda-Esperanza Esparza-Ponce

**Affiliations:** 1 Centro de Investigación en Materiales Avanzados, S. C. Miguel de Cervantes 120, Complejo Industrial Chihuahua, Chihuahua 31136, Mexico; andrea.balderrama@cimav.edu.mx (A.-S.B.-G.); claudia.ramirez@cimav.edu.mx (C.-A.R.-V.); erasmo.orrantia@cimav.edu.mx (E.O.-B.); 2 Facultad de Ciencias Químicas, Universidad Autónoma de Chihuahua, Chihuahua 31125, Mexico; lilanderos@uach.mx

**Keywords:** nanoparticles, antimicrobial resistance, bacteria, resistance mechanism

## Abstract

Antibiotics are being less effective, which leads to high mortality in patients with infections and a high cost for the recovery of health, and the projections that are had for the future are not very encouraging which has led to consider antimicrobial resistance as a global health problem and to be the object of study by researchers. Although resistance to antibiotics occurs naturally, its appearance and spread have been increasing rapidly due to the inappropriate use of antibiotics in recent decades. A bacterium becomes resistant due to the transfer of genes encoding antibiotic resistance. Bacteria constantly mutate; therefore, their defense mechanisms mutate, as well. Nanotechnology plays a key role in antimicrobial resistance due to materials modified at the nanometer scale, allowing large numbers of molecules to assemble to have a dynamic interface. These nanomaterials act as carriers, and their design is mainly focused on introducing the temporal and spatial release of the payload of antibiotics. In addition, they generate new antimicrobial modalities for the bacteria, which are not capable of protecting themselves. So, nanoparticles are an adjunct mechanism to improve drug potency by reducing overall antibiotic exposure. These nanostructures can overcome cell barriers and deliver antibiotics to the cytoplasm to inhibit bacteria. This work aims to give a general vision between the antibiotics, the nanoparticles used as carriers, bacteria resistance, and the possible mechanisms that occur between them.

## 1. Introduction

The application of scientific knowledge to manipulate and control matter predominantly in the nanoscale to make use of size and structure dependent properties, and phenomena distinct from those associated with individual atoms or molecules, or extrapolation from larger sizes of the same material [1], which consist of the ability to synthesize, manipulate, and modify materials below 100 nanometers [2,3,4,5], has postulated as nanotechnology a fundamental discipline in scientific and technological advances in different areas, such as medicine and the pharmaceutical industry, to provide solutions for various existing problems in these areas.

It is considered that a nanostructured material must have dimensions within 1 to 100 nm [5,6]. However, in medicine, these values can range up to 200 nm in diameter [7]. Among these materials, the use of nanoparticles (NPs) stands out metallic, bimetallic, metal oxide, and magnetic [8,9,10].

The use of metallic and metal oxide NPs has been increasing due to the chemical and physical intrinsic properties acquired by NPs synthesized [11]. Depending on their application, the optical, catalytic, and electrical behavior, mechanical and chemical stability [12,13], as well as morphology and particle size, can be controlled [14], which makes them suitable for the pharmaceutical industry.

Metallic NPs allow the possibility of interacting at biomolecular levels [15]. This improves detection, treatment, and monitoring of pathologies, through the specific targeting of cells and tissues. In addition, it helps with the administration of drugs, evaluation of diseases, and treatment of degenerative conditions [16], which makes them promising materials for directing chemotherapeutic drugs.

The pharmaceutical and medical industry was presented with one of the biggest problems worldwide since 2015, when the World Health Organization (WHO) declared the increase of antimicrobial resistance by pathogenic bacteria as a priority to study. The WHO published in 2017 a list of pathogens with the highest risk worldwide (Table 1) [17]. These bacteria are resistant to antibiotics and have been classified based on various criteria, such as mortality and resistance prevalence, among others, classifying them as critical, high, and medium priority [18].

Antimicrobials are organic small molecules (they vary in size at angstrom level) that prevent the development of pathogenic microorganisms, which are generally used in bacteria. Antimicrobial agents can be divided into three groups according to their characteristics: disinfectants, antiseptics, and those for clinical-therapeutic use [19]; the latter are known as antibiotics capable of reducing and controlling the presence of bacteria that have invaded the patient’s body.

Before the use of antibiotics, the mortality rate caused by pathogenic bacteria was high [18]. However, at the end of the 19th century and the beginning of the 20th century, antibiotics began to be studied. This led to the discovery of penicillin, using it clinically in 1930, together with sulfamide. These antibiotics were effective against Gram-positive and Gram-negative bacteria [20]. Unfortunately, the capacity of these antibiotics to treat infectious diseases caused by bacteria has not been enough, and this represents a danger for the population [21].

Excessive and uncontrolled use of antibiotics have generated resistance to antimicrobials by bacteria, as well as the spread of resistant bacteria in hospitals, and have become some of the most important problems in recent years [22].

In the United States alone, according to the Centers for Disease Control and Prevention (CDC), the first report on threats by antimicrobial resistance was published in 2013. This report mentions that, in the U.S., at least 2 million people contract an infection by bacteria resistant to antibiotics, and at least 23,000 people died because of this [23]. However, in 2019, an increase to 2.8 million infected patients by resistant bacteria was reported, of which more than 35,000 people died every year. Thus, producing an economic impact of more than 4.6 billion dollars annually in the United States alone [24].

On the other hand, in the publication of projections made by the Organization for Economic Co-operation and Development (OECD), it is predicted that, by 2030, the increase in deaths caused by resistant bacteria will increase up to 60% in less developed countries, while the increase is approximately 5 to 20% in more developed countries [25].

In this work, we discuss some articles on metal and metal oxide nanoparticles having been used as a means of transportation of antibiotics to reduce antimicrobial resistance and also to analyze the various inhibition mechanisms. In summary, the NP size, morphology, and surface functionalization of the antibiotic, type of resistance mechanism, and nanoparticle toxicity are analyzed.

## 2. Methodology

This review was done to answer the following questions: what are synthetic antibiotics, and what is their classification? What is antimicrobial resistance, and why does it develop? What are the mechanisms of resistance of bacteria to antibiotics? What are the mechanisms of action of inorganic nanoparticles against antimicrobial resistance? What are the mechanisms of action of inorganic nanoparticles against antimicrobial resistance? In addition, how has the use of inorganic nanoparticles against antimicrobial resistance influenced the development of antimicrobial resistance? Which nanoparticles are being used to combat antimicrobial resistance? What are the mechanisms of action of inorganic nanoparticles against resistance? How has the use of inorganic nanoparticles functionalized with antibiotics influenced resistance?

We started to study articles from different official databases, such as Elsevier, ScienceDirect, PubMed, Google Scholar, Scopus, and SciFinder, to identify relevant papers according to the topic.

To describe the current situation and discuss research on inorganic nanoparticles functionalized with synthetic antibiotics, we chose to search for articles between the years 2015–2021. While, for basic information articles, such as what are antimicrobials and the types of resistance presented by bacteria, information was obtained from research, books, and review articles from 2005–2021.

The keywords used throughout the literature search were: nanoparticles, gold nanoparticles, silver nanoparticles, copper oxide nanoparticles, titanium oxide nanoparticles, zinc oxide nanoparticles, antimicrobial mechanism nanoparticles, ROS generation, antimicrobial resistance, bacteria, antibiotics, chromosomal mutation, biochemical mechanisms, pathogens, antibiotic mechanisms of action, World Health Organization, biofilm generation, and bacterial cell membrane inhibition.

A total of 300 to 400 articles were read, of which only 246 are cited because they were those that provided relevant, basic information or contributed something of importance to the review.

## 3. Antibiotics

Antibiotics are antimicrobial drugs capable of reducing and controlling the presence of bacteria that have invaded the tissues of a subject. Antibiotics are grouped into classes according to their chemical structure, effect, spectrum, and action mechanism [26,27,28,29].

### 3.1. By Chemical Structure

According to their chemical structure, the antibiotics can be grouped as β-lactam antibiotics, macrolides, aminoglycosides, and tetracycline antibiotics [30].

### 3.2. By Effect

This group corresponds to those that caused the death of the most sensitive microorganism, in the bacteria growth phase (bactericidal) or those that inhibit bacterial growth (bacteriostatic) [31].

### 3.3. By Spectrum

This classification is divided into three branches: the broad spectrum, the limited spectrum, and the narrow spectrum. When talking about broad spectrum antibiotics, it emphasizes that the drug acts on a wide range of bacteria which can be Gram-positive and Gram-negative. Limited spectrum antibiotics are those acting only against Gram-positive or Gram-negative cocci, as well as Gram-positive bacilli and spirochetes, as is the case with penicillin. Lastly, narrow spectrum antibiotics, which attack only a very small sector of bacteria [32].

### 3.4. By the Mechanism of Action

The classification of antibiotics according to their mechanism is divided into four main ones which consist of inhibiting cell wall synthesis, protein synthesis, nucleic acid synthesis, and antimetabolites.

According to Murray et al. [32], in Medical Microbiology, antibiotics for inhibiting cell wall synthesis are the penicillins, cephalosporins, carbapenems, and cephamycins, since they bind to penicillin-binding proteins (PBP) and enzymes responsible for peptidoglycan synthesis. On the other hand, vancomycin, such as the other antibiotics, usually damages the cell wall; however, the mechanism of vancomycin is to inhibit the cross-linking of the peptidoglycan layers, such as cycloserine, thus causing cell death. Bacitracin is responsible for inhibiting the cytoplasmic membrane of the bacterium, as well as the movement of peptidoglycan precursors. Antibiotics of the polymyxin family often damage the bacterial membrane [27].

In the case of inhibiting protein synthesis, in Murray et al. [32], drugs, such as aminoglycosides, are used because they are responsible for the premature release of peptide chains from the 30S ribosome. Likewise, tetracyclines damage proteins by preventing polypeptide elongation in the 30S ribosome. Antibiotics of the macrolide, ketolide, clindamycin, oxazolidinone, and streptogramins groups are responsible for preventing protein synthesis and polypeptide elongation on the 50S ribosome.

The groups of quinoline, rifampicin, rifabutin, and metronidazole are antibiotics that usually impair nucleic acid synthesis, i.e., their mechanism of action is given by binding the DNA gyrase subunit, preventing transcription by binding DNA dependent RNA polymerase [32].

Finally, according to Murray et al. [32], antibiotics of the sulfonamide, dapsone, and trimethoprim families are responsible for damaging the metabolic pathways of bacteria, as they tend to inhibit dihydropteroate synthase and dihydrofolate reductase, which triggers the folic acid synthesis disruptions.

## 4. Antimicrobial Resistance

Antimicrobial resistance (AMR) is a natural phenomenon of bacteria [33] that develops thanks to its intrinsic evolutionary nature, as well as its easy and rapid adaptability to various environments [34]. However, the abuse and excessive use of antibiotics has given bacteria the ability to create greater resistance to antimicrobials [35,36,37,38,39,40,41,42], which translates into the lack of ability of antibiotics to inhibit the growth of pathogens [43]. This has alerted public health organizations worldwide and has led to major regulated and controlled antibiotic administration measures, to improve treatments in patients [44,45].

Resistance levels can vary greatly according to the groups of bacteria. Susceptibility and resistance are generally measured as a function of the minimum inhibitory concentration (MIC), which is the minimum concentration of the drug that will inhibit bacteria growth [46]. Susceptibility is a range of the average MICs for any given drug in the same bacterial species. If the average MIC for a species is in the resistant part of the range, such species are considered to have intrinsic resistance to that drug. Bacteria can also acquire resistance genes from other related organisms, and the level of resistance will vary according to the species and the genes acquired [47,48].

When referring to intrinsic resistance, it means that bacteria can be naturally resistant to some antibiotics [49], and this is due to the particular characteristics of each bacterium, which depend on its structure and function [50]. That is when the composition and chemical structure of the antibiotic is unable to penetrate or react with the structure of the bacterial membrane. An example of this type of resistance is *Pseudomonas aeruginosa* because it has a membrane with low permeability, and this makes it naturally resistant to most antimicrobials [51,52,53,54].

On the other hand, bacteria can also acquire various AMR mechanisms either by gene transfer mechanisms or by biochemical mechanisms [55]. Among the genetic mechanisms are the chromosomal and extra chromosomal mutation [56], which is called acquired resistance [57]. This type of AMR is due to the evolutionary pressure that bacteria develop against the attack of antibiotics, changing their genome through genomic mutation or by cellular selection. This exchange of genes is carried out through transformation, transduction, or conjugation [32].

Mutations develop after excessive exposure to antibiotics, which provides pathogens with strong resistance mechanisms and, therefore, greater virulence, which complicates drug treatment against bacterial infections and can result in a greater complication [48].

The biochemical mechanisms of AMR can occur due to the modification of the antibiotic bacterial target, but the enzymes that modify antibiotics are only capable of affecting certain antimicrobials [55,58]. The enzymatic inactivation of antimicrobial drugs is when there are mutations in genes that can encode porin proteins around the bacterial membrane to slow down the action of antibiotics. Another biochemical mechanism is the flow pump system that can expel antimicrobial drugs without being damaged and the reduction of intracellular concentrations because of the decrease in permeability and flow [59,60].

AMR can be caused not only by chromosomal or extrachromosomal mutations but also by cross-transfer. This means that a bacterium resistant to one antibiotic or a family of antibiotics, in particular, when encountering another antibiotic or another group of antibiotics with a similar chemical structure, will likely recognize such structure and create this immunity to this new family of antimicrobials [61,62,63].

### 4.1. Mechanisms of AMR Gene Transfer

Two types of AMR gene transfer exist: chromosome mutation and extra chromosomal mutation.

#### 4.1.1. Chromosome Mutation

This type of resistance occurs when changes are produced in the genomic sequence of bacteria, specifically in the main chromosome, and it is presented by vertical transmission, i.e., they are transmitted through offspring [62]. This type of mutation appears spontaneously and is irreversible, resulting in changes in the bacterial chromosome due to various factors that can be chemical and physical or both, which leads to changes in the bacterial cell [53] that modifies the permeability and drug target to prevent the effect of antibiotics on bacteria.

The chromosomal mutation depends on whether there are changes in the suitability or virulence of the pathogen and whether these genetically modified microorganisms prevail or arise more frequently; consequently, they would begin to replicate and would continue to cause pathologies [60].

#### 4.1.2. Extra Chromosomal Mutation

Extra chromosomal resistance is when transmission of genetic material occurs through plasmids, transposons, and integrons [64], which are extra chromosomal material. This type of resistance is also known as horizontal genetic transmission mutation [58,65,66,67,68].

According to the National Human Genome Research Institute (NHGRI), plasmids are small circular DNA biomolecules that contain small groups of genes, which are generally associated with genes resistant to antibiotics [69]. These molecules can be separated from the chromosome and can be replicated independently of the chromosome; likewise, plasmids can be transferred between different bacterial cells [70,71,72]. Plasmids are generally responsible for developing enzymes that inactivate antibiotics [62,73,74].

On the other hand, transposons are sequences present in the genome that show a high recombination and mobility capacity, which means that they can be easily integrated into the bacterial genome [75]. These can be transferred from one plasmid to another, or from a plasmid to a chromosome, and vice versa [76]. However, unlike plasmids, transposons are not capable of self-replicate [47]. The transposition process is catalyzed by an enzyme called transposase which is encoded by the genetic element itself; for example, in Gram-negative bacteria, various transposable elements will play a crucial role in the dispersal of resistance, and some will also contribute to the mobilization of integrons [61].

Through these transposons, the AMR genes may be transferred from one bacterium to another. An important characteristic of these is that they present extra genes that encode at least one function that changes the phenotype of the recipient cell in a predictable way, such as AMRs [77].

In the case of integrons able to encode cassettes of AMR genes specialized in capturing and expressing genes that encode the integrase enzyme [78], such integrons are responsible for recognizing the exogenous gene and integrating it at points of the integron [79]. These integrons also present sites specific for recombination where they can integrate genes and the promoter to express the integrated sequences [52,80,81,82,83].

**Figure 1:**
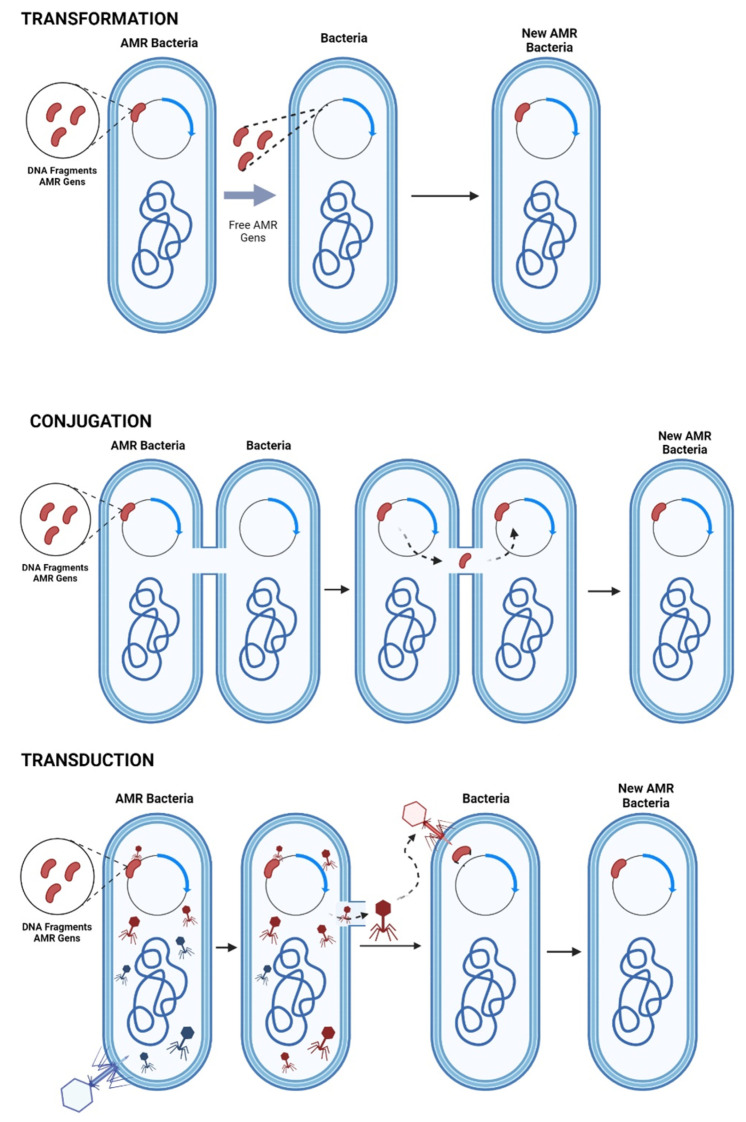
Extra chromosomal mutation: transformation, conjugation, and transduction. Created with BioRender.com (accessed on 17 November 2021).

What these genomic mutation methods have in common are the mechanisms of AMR gene transfer which are presented as transformation, conjugation, and transduction (Figure 1).

Transformation.

The transformation occurs when the bacterium can capture the exogenous from the DNA and manages to incorporate it into its genome through recombination. This process takes place in some bacteria that are from the same species; therefore, the DNA has a certain resemblance and homology [84].

Conjugation.

The mechanism of genetic transfer between two bacteria employing pili is known as conjugation; generally, by this mechanism, resistant plasmids are transferred [85].

Transduction.

Transduction occurs when a virus is capable of infecting bacteria, where said virus can transfer genetic material. The best-known strains to which this type of mechanism is attributed are *Staphylococcus* spp. [86].

### 4.2. Biochemical Mechanisms of AMR

The bacteria have four AMR biochemical mechanisms which are focused on inactivating the antibiotic and protecting its structure.

#### 4.2.1. Inactivation of the Antibiotic

This mechanism can use enzymatic inactivation, which is responsible for modifying existing cellular enzymes that react with the antibiotic [87], thus avoiding cell damage [88]. An example is β-lactamase enzymes, which can hydrolyze the most common antibiotics, such as penicillins and cephalosporins. Likewise, bacteria can inactivate antibiotics through the transfer of acetyl, phosphoryl, and adenyl chemical groups into the antimicrobial drug, with acetylation being one of the mechanisms best known for the inactivation of aminoglycosides and chloramphenicol, among other groups of antibiotics [89].

#### 4.2.2. Antibiotic Excretion

**Figure 2:**
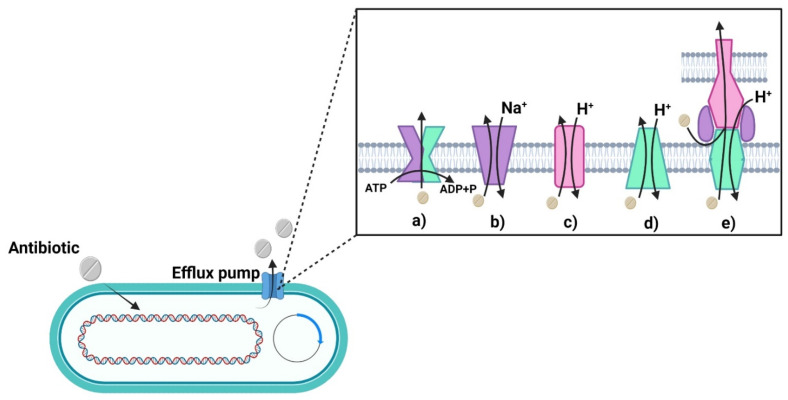
Efflux pump families: (**a**) ABC, (**b**) MATE, (**c**) SMR, (**d**) MFS, (**e**) RND. Created with BioRender.com (accessed on 17 November 2021).

Another way to avoid cellular damage caused by antibiotics is the excretion of antimicrobial drugs through the activation of outlet pumps [90], which are proteins that can eliminate or get rid of a wide variety of antibiotics and compounds from the periplasm to the outside of the cell [91]. They are outlet pumps responsible for eliminating all toxic substances for bacteria, preventing their death. Five main families of pumps have been observed, which are classified according to their structure and the available energy source (Figure 2) [62,92].

The ABC Family handles ATP-binding cassettes, through the transport of amino acids, drugs, ions, polysaccharides, proteins, and glucose [93,94,95].

The MATE Family or of extrusion of toxic compounds through Na+ used as an energy source. This type of pump can eliminate efflux cationic dyes, fluoroquinolone antibiotics, and some aminoglycosides [96,97,98].

The SMR Family is a group of small resistance to multiple antibiotics, which uses the energy of the H+ protons, and, because these have hydrophobic nature, these can expel lipophilic cations [99,100].

The MFS Family are facilitator superfamily pumps that can dispose of antibiotics through the transport of anions, metabolites, and glucose [101,102,103].

The RND Family is given by resistance nodulation cell division, as these can catalyze the flow of substrates through the substrate/H+ anti-port mechanism. This type of pump can not only get rid of antibiotics but other antimicrobials, such as detergents, heavy metals, solvents, etc., that compromise the life of the bacteria [104,105].

#### 4.2.3. Permeability of the Outer Membrane

This mechanism is given by generating changes in the lipid bilayer regardless of whether the permeability of the membrane is altered by changes in the porins, i.e., nothing can be absorbed, resulting in the entry of small molecules, such as antibiotics, to be limited. Likewise, some bacteria have managed to generate biofilms that enter into this resistance mechanism [106], where they manage to create through the same colony a kind of shell that protects them through different biomolecules to prevent the antibiotic from penetrating the membrane [18,107,108]. These biofilms are made up of lipids, polysaccharides, proteins, and extracellular DNA, which are responsible for interacting with antimicrobial agents, whether antibiotics or nanoparticles, modifying surface charge, size, concentration, and particle shape in the case of NPs, while, in the case of antibiotics, these are capable of modifying the chemical structure [109,110,111].

#### 4.2.4. Target Modification

This type of mechanism is characterized by modifying or generating changes in the structures of antibiotics in specific places or in the target, which causes the inactivation of the drug. This mechanism takes place when the bacteria can alter the site where the antibiotic binds with it to deactivate the main function of the antimicrobial [112].

## 5. Nanotechnology Applied to Antimicrobial Resistance

Nanotechnology currently plays a key role in scientific and technological advances in medicine and the pharmaceutical industry, this concerns the use of materials controlling their size and shape [2]. In these senses, the nanoparticles (NPs) are particulate materials on a nanometric scale that allow modifying both the physical and chemical properties of materials, as well as their morphology and size, which ranges from 1 to 100 nm [113,114,115]. The smaller and more spherical the NPs are, the greater the surface-volume ratio is achieved, which helps to enhance the chemical and biological activities of the NPs [116,117].

The NPs which have been used for different applications [118,119], such as drug administration, photo ablation therapy, biological imaging, applications in biosensors, and even as an alternative to reduce antimicrobial resistance, have stood out with great relevance [120,121,122].

These applications include the use of NPs as antimicrobial components on advanced materials for medical devices as catheters walls, valves, stents, and a surface that could be found inside or outside the body or could be designed for in vivo therapies. The development of advanced materials includes the use of Fe_3_O_4_ functionalized with chitosan and lysozyme to produce a coating for producing biofilm-resistance surfaces [123]. The NPs designed for in vitro applications include their use as a drug administration; in these applications, the NPs can load with different molecules as an essential oil, such as the ZnONPs have been loaded with *Citronella* essential oil [124] or Oxide-Silica Core-Shell with essential oil [125], both with antimicrobial activity. In addition, Fe_2_O_3_NPs have been used as carrier paclitaxel and β-cyclodextrin [126] or PdNPs capping with polyvinylpyrrolidone load with quercetin [114], and Silica Core-Shell Au [127] for Cancer Therapy.

NPs can be classified as metallic, metal oxide, bimetallic, and magnetic [8,128,129]. It has been demonstrated that this type of particle can obtain antimicrobial properties so that, when increasing the surface area of the particles, a greater contact area with microorganisms is generated [15,130,131,132], thus enhancing its antimicrobial activity.

**Figure 3:**
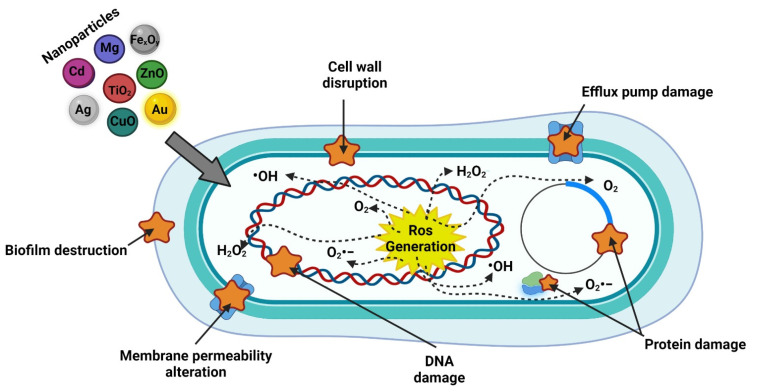
Action mechanism of inorganic nanoparticles. Created with BioRender.com (accessed on 17 November 2021).

As well as acting as antibacterial agents that can cause alterations in the bacterial membrane, metallic, bimetallic, and metal oxide NPs usually produce reactive oxygen species (ROS) by releasing metallic ions that alter the cellular components of bacteria [133,134], and the smaller nanoparticles are the damage created by them will be greater because they tend to be better absorbed on the bacterial surface. This is because, in some cases, the NPs have a positive surface charge that facilitates the union with the negative charge on the surface of bacteria [129,135,136]. Likewise, the photodynamic and photothermic effects of NPs generate a greater impact as antimicrobial agents (Figure 3), which is directly related to the release of metallic ions and ROS [6].

NPs sizes are largest than antibiotics which allow the use of these as carriers of antibiotics or other small molecules as antibodies or chemotherapeutic agents [126].

### 5.1. Nanoparticles Antimicrobial Effects

There are six main antimicrobial effects of nanoparticles: (1) Interaction with cell wall and membrane, (2) Generation of ROS, (3) Penetration of the cell membrane, (4) Inhibition of protein synthesis and DNA damage, (5) Damage to metabolic pathways, and (6) Biofilm inhibition (Figure 3) [137,138,139,140].

#### 5.1.1. Interaction with the Cell Wall and Membrane

Cell membrane and cell wall are one of the main resistance barriers that bacteria have, which are constituted by several molecules that help the adsorption of nanoparticles.

In the case of Gram-positive bacteria, the main component is teichoic acid, which causes the NPs to be distributed along the phosphate molecule chain, thus preventing their aggregation [141]. However, the fact that Gram-positive bacteria have a thick peptidoglycan wall and pores allows the penetration of smaller molecules that can cause damage to the cell wall, as well as the death of the bacteria [142].

On the other hand, in Gram-negative bacteria, this is the opposite, since having a higher concentration of lipopolysaccharides, lipoproteins, and phospholipids allows the bacteria to have a negatively charged cell wall and, thus, be able to attract NPs with greater intensity. However, these bacteria are the most prone to generate a barrier that prevents the penetration of small molecules [143,144].

#### 5.1.2. Generation of ROS

The main mechanism by which nanoparticles can damage bacteria is through oxidative stress caused by ROS because, under normal conditions, bacteria can maintain a balance in the generation of ROS. However, when in contact with some NP, this balance is affected. This causes an excess of ROS that will inevitably in an alteration of the oxide-reduction state of molecules that will favor cellular oxidation.

Main Types of ROS:

There are four main types of ROS: radial hydroxyl (·OH), singlet oxygen (O_2_), superoxide radical (O_2_·−), and hydrogen peroxide (H_2_O_2_) [145,146]. For both H_2_O_2_ and O_2_·−, there have been reports that these come from the stress of short-term reactions and are reduced by antioxidants, such as catalases. Therefore, when it comes to physiological damage, it can be attributed largely to oxidative stress caused by O_2_ [147].

#### 5.1.3. Penetration of the Cell Membrane

Once the nanoparticles manage to penetrate the cell wall, they tend to release ions and generate ROS by diffusion. In the case of the release of metal ions, one of the main mechanisms that have been observed is the affinity of the ions to bind to the negatively charged functional groups of the cell membrane, such as phosphate and carboxyl groups. This phenomenon is known as adsorption [141,148,149,150].

#### 5.1.4. Inhibition of Protein Synthesis and DNA Damage

Another of the most reported mechanisms attributed to metal nanoparticles is DNA damage and inhibition of protein synthesis. These usually cause a breakdown in the ribosomal subunit proteins, enzymes, and other proteins synthesized in the membranes of the bacterial cell. Likewise, a degradation, compression, and fragmentation of bacterial DNA have been observed, resulting in a reduction of the physiological activity of genes [151,152].

This was demonstrated in a study by Su et al. [153] using ZnONPs against *E. coli* DNA. They found that concentrations and the greatest damage caused by the NPs were found in 10 areas of the bacterial genome, as well as gene expression, ribosome composition, molecular structure-activity, and RNA modification, were altered in the presence of the NPs.

Similarly, Nagy et al. [154] used silver NPs to cause DNA damage by positively regulating various antioxidant genes, metal depletion, ATPase pumps, and genes encoding metal transport in *S. aureus* and *E. coli*. In this study, they concluded that silver nanoparticles have an antibacterial mechanism that causes a depletion of the antioxidant capacity of bacteria.

#### 5.1.5. Damage to Metabolic Pathways

Metabolic pathways of bacteria are not isolated and are integrated in a complex way in the activity of the cells since their main role is to maintain the growth and reproduction of bacteria. However, it has been observed that, when nanostructures enter the bacterial cell, alterations in metabolism occur, causing damage to the cell membrane, inducing oxidative stress and, finally, the death of the bacteria [155,156]. It has also been proposed that NPs can regulate and damage the metabolic processes directly of the target proteins of the bacteria, which is why they can affect the adhesion of bacteria and the formation of biofilms [155].

#### 5.1.6. Biofilm Inhibition

One of the main mechanisms that nanoparticles present when interacting with biofilms generated by bacteria is the interaction with EPS, which will allow the access of any chemical molecule agentive to the bacteria and, thus, cause damage to the cell [157,158]. It has also been reported that NPs in contact with bacteria can affect the bacterial adhesion rate causing damage to biofilms, which is attributed to metabolic inhibition processes by releasing metal ions; however, the specific mechanisms cannot yet be fully explained.

### 5.2. Interaction between Antibiotics, Nanoparticles and Bacteria

The inorganic nanoparticles most used to inhibit the growth of bacteria due to their antimicrobial properties are those made of gold (Au), silver (Ag), silicon (Si), iron (Fe), silver oxide (Ag_2_O), copper oxide (CuO), titanium oxide (TiO_2_), zinc oxide (ZnO), and magnesium oxide (MgO) [2,8,119]. However, not all of the NPs are usually used for medical applications because they could have toxicity [138,139,159]. In addition, they can be accumulated on different organisms [160,161] and tissues [162]. Such is the case of apatite NPs in lipids that surround the site where the NPs was collocated. As well, the NPs were preferentially taken up by macrophages [163]. The mice exposition to AuNPs showed an increase in the Au accumulation after 6 h and liver and spleen accumulation of it [164].

The use of NPs together with antibiotics have been proved to act in a synergic way [124,165], reduce the dose used of antibiotic and NPs, achieve a high local concentration [166] or inclusive reverse the antibiotic resistance [165,167].

As with antibiotics, NPs also have various mechanisms of action against bacteria because they can alter the metabolic activity of pathogens. It has been observed that NPs act when they are in contact with the bacterial cell walls, and, because of this, the following interactions have been proposed to explain how the contact between the NP and the bacteria is: electrostatic attraction, by ligand-receptor interactions, hydrophobic reactions, and Vander Waals forces [168,169,170,171].

Likewise, there are metallic ions that are released through the metal oxides that are to be absorbed in the membranes of the bacteria, allowing them to interact with the functional groups of biomolecules, such as proteins and nucleic acids. This will trigger direct changes in the structures of bacteria, as well as the overproduction of enzymes, which will generate physiological disturbances [172,173,174].

It has been observed that bacteria produce an extracellular matrix that is responsible for nanoparticles agglomeration. This generates bacterial resistance to nanoparticles with a size larger than 10 nm [175,176]. Regarding nanoparticles smaller than 10 nm, bacteria have been mutating their genes, achieving to make changes in the regulation of reduction of porins, which prevents NPs from entering the cells.

Such was the case of *P. putida* which was able to change the composition of unsaturated fatty acids found in the membrane, generating a less permeable membrane [177].

The same happens when bacteria are attacked through the surface charge of both the nanostructure and the bacterial membrane because bacteria are capable of regulating and modifying the electrical charge of their surface, which will cause the nanoparticles to be repelled [178,179]. According to Niño-Martínez et al. [157], this phenomenon is supported by the envelope stress response (ESR) mechanism present in Gram-positive and Gram-negative bacteria. This one is responsible for monitoring biogenesis, as well as protecting the integrity of the bacterial envelope.

Unlike antibiotics, NPs have dimensions smaller than 100 nm, resulting in novel physicochemical properties which can have greater interaction with cells due to a higher volume-to-surface ratio, making them versatile for strategic adjuvants. The mechanisms of action of antibiotics are usually relatively basic and simple, resulting in genomic mutation of bacteria to resist their mechanisms. However, NPs alone often have complex mechanisms that act simultaneously to prevent the generating genomic mutations of bacteria and inhibit their growth.

NPs used as transport media, loaded or functionalized with antibiotics, can enhance the mechanisms of action of the drug. This is because the particle size >100 nm is so small that the bacterial phagocytes can easily phagocytose them, in addition to the fact that the morphology of the particle itself allows greater flexibility to penetrate the cell and cause endocytosis, allowing the drug to be released intracellularly [180].

Another advantage of NPs as an antibiotic adjuvant is that they function as protectors, which means that the NPs can increase the serum levels of the drugs and, in this way, can protect from the enzymatic action of the target [181].

Likewise, by having an NP functionalized with an antibiotic, a more controlled and potent administration of the drug can be obtained, activating the effect of the NP through controlled stimuli of light, pH, photothermal, and magnetic, among others, which, unlike the antibiotic, would only need to be exceeded in doses and repeatedly to achieve the same effect [182,183].

The efficiency of antibiotics gets lower as time passes, and additionally, the human body can only absorb 50% of the antibiotic, while the other 50% is excreted in the urine [184], which further lowers its efficiency. It has been reported in the literature how complicated it is to stimulate the absorption of antibiotics in high doses due to the toxicity that the drug can present in the organism, as well as the development of side effects in the patient [185].

Such was the case of the research proposed by Qi et al. [185], where they explain that vancomycin has a strong mechanism of action against Gram-positive bacteria; however, the level of toxicity it has in the organism is high, causing side effects in kidneys and ears. Therefore, they proposed the synthesis of mesoporous silica nanoparticles functionalized with vancomycin with which they were able to inhibit the cell growth of Gram-positive pathogens selectively in macrophage-like cells.

For this reason, the targeting of antibiotic-functionalized nanoparticles employing an active targeting, which can be magnetic or by temperature, has been used. Table 2 shows some studies reported by different researchers, in which a greater inhibition of multi-resistant bacteria is reported when using metal nanoparticles functionalized with antibiotics.

In the next sections, a compilation of works with different compositions of NPs for inhibition of bacteria are analyzed.

#### 5.2.1. Silver Nanoparticles

Silver nanoparticles (AgNPs) have become one of the most studied protagonists for the inhibition of bacteria due to their high antibacterial properties in concentrations that are not cytotoxic for humans, and they have become strong candidates to replace antibiotics for clinical use against bacterial resistance.

The mechanisms of action of AgNPs as antimicrobial agents depend on physicochemical properties [186], that is, on their morphology, size, whether they are linked or functionalized with any biomolecule or metal [177,187]. However, it has been reported that one of the main mechanisms of AgNPs is their binding to the cell wall and membrane, damaging biomolecules and structures found within the cell, as well as oxidative stress causing the release of silver ions [188,189,190,191].

The use of silver as an antimicrobial agent dates back to 1852 when it was used by Dr. J. Marion Sims for the treatment of vesicovaginal fistulas. In 2007, Pal et al. [192] studied the antimicrobial effects of silver by varying particle size, obtaining results that show that, by decreasing the particle size, it was possible to increase the surface area, which would result in a greater affinity when interacting with the biomolecules in bacterial cells. They observed that the morphology of the particle had a great influence to enhance the antimicrobial activity, thus concluding that the triangle-shaped nanoparticles generated a greater cell death compared to the ones with a spherical shape or nano-rods.

This was also confirmed by Nanda et al. [193] as they conducted a study about the biosynthesis of AgNPs with *Staphylococcus aureus* and its antimicrobial activity against Methicillin-Resistant *S. aureus* (MRSA) and Methicillin-Resistant *Staphylococcus epidermis* (MRSE), including Methicillin-Resistant *Streptococcus pyogenes*, *Salmonella typhi*, and *Klebsiella pneumoniae*. They reported AgNPs of approximately 160 to 180 nm and of irregular shape, which was characterized by the AFM technique. In the toxicity trials, for the inhibition of the bacteria they used a concentration of 20 µL (0.002 mg) of AgNPs, bacteria Gram-positive was the affected bacteria. Was observed a diameter reduction of the bacterial cultures to 18 mm for MRSE, 17.5 mm for MRSA, and 16 mm for *S. pyogenes*. They concluded that the susceptibility for Gram-positive bacteria, and especially for MRSA bacteria, is because of the inhibition of the synthesis of the bacterial cell wall.

In 2010, Lara et al. [194] conducted a very similar study in which they sought to inhibit the growth of the pathogens *S. pyogenes* resistant to erythromycin, *E. coli* 0157:H7 resistant to ampicillin, and *P. aeruginosa* resistant to multiple antibiotics, using a concentration of 6.25, 12.5, 25, and 50 mM of AgNPs of 100 nm. They reported the inhibition of 99.7% of *S. pyogenes*, 95.7% for *E. coli*, and *P. aeruginosa* 92.8%, concluding that the higher the concentration, the greater the death of bacteria, and also confirming that time is not an important factor for AgNPs to cause bacterial inhibition. However, they decided to carry out studies to see if bacteria could become resistant to the AgNPs functionalized with antibiotics, leaving the bacteria exposed to the AgNPs for 3 weeks. They reported that MRSA could grow at a concentration of nanoparticles of 100 mM, *S. aureus* at 200 mM, and, finally, for *P. aeruginosa* and *E. coli*, they achieved to generate resistance at the concentration of 75 mM. These results show that lipopolysaccharides could trap and block positive charges of silver nanoparticles and make Gram-negative bacteria less vulnerable to nanoparticles. However, it has also been proposed that AgNPs adhere to the surface of the cell membrane, thus altering their function, penetrating the cells, and releasing silver ions that cause oxidative stress through ROS. On the other hand, resistance to AgNPs implies changes in the inhibited cell target, so, if there is a change in proteins or in how antibiotics are directed, the bacterial sensitivity to the antibiotic used can be modified.

In 2018, Panáček et al. [179] demonstrated the resistance of the Gram-negative strains *E. coli* and *P. aeruginosa* to AgNPs after repeated exposures. They determined the minimum inhibitory concentrations (MIC) of AgNPs at 432 mg/L. Their results focus on bacteria repeatedly exposed to sub-inhibitory concentrations of AgNPs, where pathogens were able to rapidly develop AMR. They state that said resistance is due to the production of flagellin, which is an adhesive protein of the bacterial flagellum that causes the aggregation of AgNPs, eliminating its antimicrobial effect against Gram-negative bacteria.

Ashraf et al. [195], in their study of bacterial extracellular proteins interacting with silver ions for the production of AgNPs encapsulated in proteins, found the potential that the *E. cloacae* protein has within the synthesis of AgNPs. It assists in the elimination of the risk of toxic agents, and this could have a great impact on medical applications, since it generates greater biocompatibility. In this study, they carried out various nanoparticle’s synthesis by chemical and biological methods, where they obtained nanoparticles with a size approximate of 58 nm, to which they exposed various Gram-positive and Gram-negative strains, proposing the mechanism of interaction between the extracellular protein and the silver ions that are released to cause cell death. However, they emphasize the need for more research to confirm their hypothesis.

The discovery of antibacterial and catalytic activities of AgNPs that are biosynthesized with *Convolvulus fruticosus* (CF-AgNPs) to inhibit the growth of pathogens resistant to multiple drugs was studied by Shirzadi-Ahodashti et al. [196]. They obtained nanoparticles of 45 nm with spherical morphology. AgNPs have been exposed to the strains *S. aureus*, *E. faecalis*, *A. baumannii*, *E. coli*, *P. mirabilis*, *K. pneumoniae*, and *P. aeruginosa*, using a MIC of 0.1 µg/mL for *S. aureus*, *A. baumannii*, and *E. faecalis*, which had a greater inhibitory response compared to the exposure of the reference antibiotic ciprofloxacin, while *E. coli* showed the same inhibitory activity for both the antibiotic and AgNPs. Finally, *K. pneumoniae* and *P. mirabilis* showed a lower sensitivity to CF-AgNPs with MIC. The authors report various mechanisms of action of nanoparticles, such as the release of lipopolysaccharides, electrostatic interactions, and alterations in the permeability of the cell membrane. However, they specifically describe that a binding and penetration of CF-AgNPs into the bacterial membrane was observed through the destruction of the cell wall; likewise, some reactions occurred with the thiol groups (-SH) of proteins, and, finally, DNA replication was prevented, causing bacterial death.

#### 5.2.2. Zinc Oxide Nanoparticles

Zinc oxide nanoparticles (ZnONPs) have been of interest to researchers because they are inorganic semiconductors, which can be easily absorbed by organisms in regulated concentrations [197]. It has caused the fact that the research of ZnONPs focuses on environmental, biological, cosmetic [198], and renewable energy applications [199], such as catalysts [200], biosensors, and even microbial enzyme inhibition, among many other applications. Its application in the medicine and pharmaceutical industry is not behind because, recently, its use as anti-inflammatories, for drug administration, cancer therapy, and as antimicrobial agents [201] on their own or as a potentiator for antibiotics [202] has been studied.

As in other metal or metal oxide nanoparticles, ZnONPs have mechanisms of action against bacteria. However, their exact mechanism remains difficult to confirm. For this reason, various authors have supported the interactions caused by ROS, since inducing oxidative stress in cells interrupts the synthesis of biomolecules, such as lipids, proteins, and even DNA, resulting in bacterial death [173,203]. In addition, it has been proposed that particle size, as well as the morphology they present, can cause less or more damage, depending on what is intended, since these can enter the cells and damage the integrity of bacteria by attacking the membrane [202,204]. Finally, there is a discussion about the possibility that Zn^+2^ ions can delay the growth of bacteria by binding with the receptors located in the membranes [205,206,207].

In 2010, Banoee et al. [208] conducted a study of ZnONPs where they improved the antibacterial activity of the antibiotic ciprofloxacin in *S. aureus* and *E. coli* bacteria. The authors report nanoparticles of 10 and 45 nm in diameter with a concentration of 500 µg and obtained an increase in the zone of inhibition by 27% for *S. aureus* and by 22% for *E. coli*. They concluded that ZnONPs are powerful adjuvants for antimicrobial inhibition when working together with antibiotics because the mechanism of action of antimicrobials is potentiated.

Patra et al. [209] carried out the study of ZnONPs with a size of 18 to 20 nm and with a semispherical morphology, which was functionalized with the antibiotics ciprofloxacin (CIP), whose ligands were verified by the FTIR spectroscopic technique. Inhibition of *S. aureus*, *Klebsiella* sp., and *E. coli* was given with a MIC of 10 µg/mL of ZnONPs-CIP, obtaining favorable results for bacterial death compared to the trial carried out of the pathogens with the antibiotic. The authors reported that the combination between the nanoparticle improve the antibiotic activity, causing damage to the bacterium cell membrane, which allowed ciprofloxacin to enter into the cell, causing ROS and, thus, interrupting cell division.

The biocompatibility of ZnONPs has been demonstrated, Zhong et al. [210] incorporated ZnONPs into carboxy methyl chitosan (CMCS) by spray drying. They demonstrated that both ZnONPs and CMCS-ZnO microspheres of sizes from 1 to 6 µm in diameter had the same antimicrobial activity, which was dose dependent. The activity against *E. coli* showed that the ZnONPs alone have better activity with the smallest NPs (10 nm). These NPs exhibit an 87% of inhibition with 125 µg/mL while the ZnONPs with a size of 30 nm, while ZnONPs with a size of 10 nm showed a 97% of inhibition with 62.5 µg/mL. The use of CMCS-ZnO microsphere showed an increase of concentration for the *E. coli* inhibition until 2 µg/mL. The explanation for this is that the CMCS-covered ZnONPs turned out to have a better interaction when binding to the cell membrane through the –NH_2_ group of the CS group, thus, improving the permeability of the membrane and making the cytoplasm capable of filtering the nanostructure causing cell death.

The ZnONPs antimicrobial mechanism is based on their ability to damage the integrity of the cell membrane, slow down the replication of AMR genes in bacteria, prevent the formation of biofilms, and decrease the hydrophobicity of the cell surface. Likewise, the antibacterial action of these nanoparticles depends significantly on their size, since it is a crucial factor due to the ease of entry of small particles through the pores on the surface of the bacterial cell. These pores on the surface of the bacterial cell are in the nano-size range. Furthermore, the ZnONPs exhibited anticancer activity compared to normal cells. Two mechanisms based on ROS production, ZnONPs toxicity and induction of apoptosis, were predicted [211,212,213,214].

#### 5.2.3. Gold Nanoparticles

AuNPs arrived at the pharmaceutical and medical industry due to their intrinsic properties because they can be synthesized in different sizes and morphologies. In addition, the reduction from Au^+3^ to Au^0^ facilitates their functionalization with ligands, such as aptamers, polymers, drugs, and genetic material, among others [215,216,217]. In addition, the chemical inertness of gold allows good in vitro and in vivo biocompatibility [218], so there has been a proposal to functionalize AuNPs surfaces with antibiotics to increase antibacterial efficacy against resistant pathogens [219].

In 2016, a new approach was reported using gold nanoparticles functionalized with chitosan streptomycin (CANP). These ligands were characterized by various techniques, such as UV-Vis, SEM, TEM, and DLS. The 35 nm size spherical nanoparticles were studied to avoid the formation of biofilms in microorganisms, such as *Listeria monocytogenes*, *S. aureus*, *E. coli*, *P. aeruginosa*, and *Salmonella typhimurium*, obtaining favorable results for research, by inhibiting the survival of bacteria up to 95%, whose mechanism of action by the nanostructure was focused on cell wall damage [220].

Kalita et al. [167] demonstrated the increase in the bactericidal activity of the beta-lactam broad spectrum drug against Methicillin-Resistant *Staphylococcus aureus* (MRSA). In this research, they functionalized gold nanoparticles with amoxicillin (Amox) through the electrostatic interaction of the attraction forces regulated by the protonated amino and the thioether group. The AuNPs-Amox complexes were tested in in vitro and in vivo trials, where they revealed a potent anti-MRSA activity, improving the survival rate of clinical patients. They reported that the use of the nanostructure could assist the antibiotic in penetrating inside the cell. In addition, it inhibits the cell wall synthesis.

Hu et al. [221], proposed biofilms based on gold nanoparticles for photothermal ablation treatment to fight the resistance of Methicillin-Resistant *Staphylococcus aureus*. The AuNPs were 14 nm in diameter with a mixed charge of hybrid ions, which adapt to the surface of any biomolecule, and were functionalized with 10-mercaptodecyl and electrolytic 11-mercaptoundecanoic acid. The obtained favorable in vivo results, in which healthy tissues did not show damage by AuNPs due to NIR light irradiation, showing damage and inhibition of the spread of bacteria.

Due to generalized multidrug resistance caused by antibiotic abuse, Xie et al. [222] proposed the use of 2 nm diameter gold nanoparticle (AuNP) to fight multi-resistant bacteria (MDR), coating AuNP with quaternary ammonium (QA) as a solution to MDR Gram-positive bacteria, including Methicillin-Resistant *Staphylococcus aureus* (MRSA) and Vancomycin-Resistant *Enterococci* (VRE) in vivo assays. They present results where QA-AuNP kills bacteria through combined physicochemical mechanisms without causing damage to surrounding tissues in the living organism.

In 2019, a study in which there was an evaluation of the antibacterial activity of gold nanoparticles of 10 nm in diameter, functionalized with gentamicin and amikacin in *Acinetobacter baumannii* strains from patients with severe burn infections, was reported. They obtained results of 94.5% in the bacterial inhibition of AuNp with amikacin, while AuNPs functionalized with gentamicin had an antimicrobial effect 50% superior to the use of gentamicin alone. They concluded that the combination of amikacin and gentamicin with AuNPs has a very significant antibacterial efficacy against *A. baumannii* [223].

Khan et al. [224] used gold nanoparticles bound to chitosan oligosaccharide (COS-AuNPs) to inhibit the formation of *Pseudomonas aeruginosa* biofilms, where they obtained favorable results for the eradication of biofilms and were able to reduce bacterial hemolysis and different virulence factors produced by *P. aeruginosa*. They concluded that the hybrid COS-AuNPs nanoformulation could act as a potential agent to exhibit inhibitory properties against pathogenesis derived from biofilm formation as a result of a resistance mechanism of *P. aeruginosa*.

In more recent studies, Riaz et al. [225] reported the effects of gold nanoparticles coated with flavonoids (FauNP) with spherical shape and 23 nm in diameter in mice, against the resistance of *Enterococcus faecalis*, which mainly colonize tissues in the liver and the kidneys. They obtained significant results in the reduction in bacterial counts in vivo and in vitro in organs compared to free flavonoids.

Chavan et al. [166] synthesized 25 nm AuNPs coated with ampicillin (AuNPs-Amp) and evaluated their interaction with *Escherichia coli* bacterial cells. The results showed a successful accumulation of AuNPs-Amp on the surface of the bacterial cell, forming pores in the bacterial membrane. They evaluated membrane damage by atomic force and fluorescent microscopy, and functionalized particles showed promising antimicrobial activity against resistance to ampicillin in *E. coli* bacteria resistant to ampicillin with an increase of 95%.

#### 5.2.4. TiO_2_ Nanoparticles

Another clear example of materials that have been of interest to researchers is titanium oxide nanoparticles (TiO_2_NPs), due to their chemical and physical stability, as well as their strong corrosion resistance. These nanoparticles have low toxicity at low concentrations without altering their antimicrobial activity, which has makes them even more interesting. In general, TiO_2_NPs are reported in conjunction with other types of antimicrobial nanoparticles. Such is the case in the study carried out by Stoyanova et al. [226], where they prepared TiO_2_-ZnO nanocompounds to study their bactericidal properties in *E. coli* strains. Another similar case was that of Menazea et al. [227], where they studied a ZnO compound spiked with TiO_2_ to evaluate its antimicrobial effect against resistant strains *E. coli*, *P. aeruginosa*, *S. aureus*, and *B. subtilis*, obtaining results of an increase in inhibition when ZnO NPs were spiked with TiO_2_ compared to when they were each separately.

Sunscreen with TiO_2_/Zn2 and TiO_2_/Ag has been developed to inhibition of UV radiation and bacteria protection. Nanocomposite showed a correlation between concentration and inhibition efficiency. The most effective concentration to inhibit in 60–70% of *E. coli* and *S. aureus* bacteria was 100 mg/mL [228].

It has been proven that the interaction between the phospholipid presented in the bacteria membrane and TiO_2_ nanoparticles. This interaction has a direct relationship with the superficial charge and the pH. With acid pH, the nanoparticles have a positive charge that allowed the interaction with the membrane cell. In addition, UV irradiation has a greater effect on membrane stabilization, triggering oxidative stress [229].

TiO_2_ photoactivity has been increased with sodium nitrite, increasing nanoparticle ability to inhibit MRSA *Staphylococcus aureus* and kill *E. coli.* The reported mechanisms to inhibit these bacteria were the production of nitrogen reactive species as peroxynitrite, tyrosine nitration [230].

#### 5.2.5. Other Nanoparticles

The antimicrobial activity presented by magnetic iron, manganese, and magnesium nanoparticles has aroused the interest of researchers in recent years due to the damaging effect they have on bacteria by interfering with their respiratory and metabolic processes [40,211,231,232]. Likewise, Davarpanah et al. [233] mention in their study that the release of metal ions, the destruction of the cell membrane and wall, and the generation of ROS, as well as the internalization of nanoparticles in bacteria, are the mechanisms most representative of inhibiting the growth of pathogens.

In 2019, Madubuonu et al. [234] used magnetic nanoparticles to inhibit the growth of Gram-positive bacteria mainly, they said nanostructures were synthesized by the sol-gel method with sizes of approximately 71–90 nm. In this investigation they tested the cytotoxicity of the nanoparticles with a concentration of 256 to 2040 µg/mL approximately in *E. coli*, *Shigella*, *P. aeruginosa*, *S. aureus*, and *Salmonella typhi*, obtaining favorable inhibition results with Gram-positive bacteria, while, in Gram-negative bacteria, they failed to inhibit the growth, attributing it to the natural resistance of the bacteria by having a double lipid membrane.

On the other hand, cobalt oxide nanoparticles have been studied in the last three years after discovering their antibacterial properties [211,235]. In 2019, Dogra et al. [236] evaluated cobalt hydroxide and oxide nanostructures synthesized by the microemulsion method, obtaining favorable results for the antimicrobial property against the multi-resistant bacterium *S. aureus*, reporting that said bacterium presented a cellular contraction, rupture of the cell wall, and membrane, as well as a change in the morphology of the microorganism.

**Table 2:** Recent studies on metallic nanoparticles against pathogenic bacteria resistance.

Elemental Composition	Size and Morpho	Concentration (µg/mL)	Bacteria	Antibiotic	Inhibit	Mechanims	Author
AgNPs	10 nm	2.5	*P. aeruginosa*	N/A	~90%	AgNPs can enter cells and inhibits enzymatic systems in the respiratory chain, thereby altering their DNA synthesis	Salomoni et al. (2017) [4]
AgNPs	35 ± 15 nm Spheroide	0.35 0.5 0.05 8	*E. coli* *S. typhimurium* *S. aureus* *B. subtilis*	Chloramphenicol	50%	The combination of the AgNPs + antibiotic produced membrane damage	Vazquez-Muñoz et al. (2019) [237]
		0.05 0.1 16 0.12		Kanamycin	95%		
AgNPs	~26 nm Spheras	1 + 5 E 1 + 10 AMP 1 + 30 C 1 + 30 KF 1 + 2 DA 1 +30 TE 1 + 10 GEN 1 + 30 AMC 1 + 10 CFP 1 + 30 CXM	*S. aureus* *MRSA* *E. coli* *P. aeruginosa* *A. actinomycetemcomitans*	Erythromycin (E) Ampicillin (AMP) Chloramphenicol (C) Cephalothin (KF) Clindamycin (DA) Tetracycline (TE) Gentamycin (GEN) Amoxycillin (AMC) Cefpodoxime (CFP) Cefuroxime (CXM)	~80%	ROS generation and mechanism of action of antibiotic	Ipe et al. (2020) [238]
AgNPs	8–21 nm Spherical	15.62 15.62 7.8 31.25	*S. epidermis* *S. haemolyticus*	Ciprofloxacin Methicillin Gentamycin Rifampicin	0.25 mm 0.06 mm 0.12 mm 1 mm	ROS generation and enhancement	Thomas et al. (2020) [239]
Mesoporous silica	50–100 nm Spherical	426 170	*A. baumannii*	Cefepime Meropenem	11 mm 11 mm	Antibiotic mechanims	Najafi et al. (2021) [240]
AuNPs	33 ± 14 nm	2/4 1/2 1/2	*E. coli* *S. aureus* *S. epidermis*	Amoxicillin	31 mm 30 mm 19 mm	The combination of antibiotic and NPs increase the concentration of antibiotic at the site of bacterium-antibiotic interaction; in additionthe multivalent presentation of amoxicillin blockade of the bacterial efflux pump	Kalita et al. (2016) [167]
AuNPs	35 nm 200 nm	0.72 0.73	*Klebsiella pneumaniae* *A. baumannii*	Impinem Meropenem	72 mm I/35 & 48 mm I/200 73 mm M/35 & 46 mm M/200	The NPs improve the mechanism of action of antibiotic	Shaker et al. (2017) [241]
AuNPs	8 ± 2 nm	0.15 1.5	*S aureus* MRSA	Amoxicillin	85%	ROS generation by the antibiotic effect	Silvero et al. (2018) [242]
AuNPs	30 ± 20 nm Irregular	1.5	*P aeruginosa*	Amoxicillin	60–70%	Biofilm damage	Rocca et al. (2020) [243]
AuNPs	5 nm	1.18 0.23	*E. coli*	Colistin	- 6.8% fold	N/A	Fuller et al. (2020) [244]
AuNPs	25 nm	62.5	*P. aeruginosa* *S. aureus*	N/A	19 mm 15.8 mm	AuNPs have a significant inhibitory effect on bacteria, to their ability to associate with the bacteria cell wall and rupture it, as well as disrupting bacterial metabolism by interfering with bacterial DNA	Abdulazeem et al. (2021) [245]
TiO_2_NPs	64 ± 0.14 nm Irregular spheres	8–64	*P. aeruginosa*	Ceftriaxone Amikacin Ciprofloxacin Cefepime	96% 88% 80% 100%	The antibiotic in combination with the nanostructure increases the synergistic effect of an antibiotic as can inhibit the cell	Youssef et al. (2020) [246]

## 6. Conclusions

The inorganic nanoparticles composed of metals, including silver, magnetic metals, such as iron and magnesium, cobalt, zinc oxide, titanium dioxide, and gold, have been shown to possess high antibacterial activity. However, limited information is available on the in vivo antibacterial efficacy of nanostructures, their ability to inhibit pathogenic strains, and mechanisms of action. In general, metal nanoparticles have some advantages, such as a large surface area and multimodal applications. However, there are obstacles of toxicity, instability, and storage which prevent its replication and the limitation of information.

It has been proposed that, once NPs accumulate in the metabolic pathway and cross the bacterial membrane, they can interact with lysosomes, DNA, enzymes, and ribosomes, which triggers oxidative stress, changes in membrane permeability, cell electrolyte imbalances, heterologous alterations, protein inactivation, inhibition of resistant enzymes, and decoding of genomic expression.

The NPs’ toxicity depends on both the NPs and bacterial characteristics. Differences between experimental conditions make it difficult to compare results. However, NPs can interrupt the AMR and make the cells more sensitive to the antibiotic, generating a new use of the ancient antibiotic with improved characteristics.

Finally, nanoparticles not only improve the therapeutic activity of antimicrobials but also restrict the stimulation of resistance generated by bacteria. Thus, there is a need for developing simultaneous strategies to deactivate beta-lactamase, deactivating enzymes, efflux pumps, as well as generate damage to cell wall, and membrane, protein and DNA damage, change in cell permeability, and generation of ROS.

## Figures and Tables

**Table 1:** The official list of pathogen bacteria with declared priority by the WHO. Adapted with permission from WHO (permission 387722) [17].

Priority	Pathogenic Bacteria	Antibiotics for Which There is Resistance
Critical	*Acinetobacter baumannii*	Carbapenem
	*Pseudomonas aeruginosa*	
	*Enterobacteriaceae*	
	*Mycobacteria*	Carbapenem and 3rd generation cephalosporins
	*Mycobacterium tuberculosis*	3rd generation cephalosporins
High	*Enterococcus faecium*	Vancomycin and methicillin
	*Staphylococcus aureus*	
	*Helicobacter pylori*	Vancomycin
	*Campylobacter*	Clarithromycin
	*Salmonella* spp.	Fluoroquinolones
	*Neisseria gonorrhoeae*	3rd generation fluoroquinolone
Medium	*Streptococcus pneumoniae*	
	*Haemophilus influenza*	Non-sensible to penicillin
	*Shigella* spp.	Ampicillin and fluroquinolones
